# Aberrant Interhemispheric Functional Connectivity in Diabetic Retinopathy Patients

**DOI:** 10.3389/fnins.2021.792264

**Published:** 2021-12-16

**Authors:** Song Wan, Wen Qing Xia, Yu Lin Zhong

**Affiliations:** ^1^Department of Ophthalmology, Jiangxi Provincial People’s Hospital, Nanchang, China; ^2^Department of Endocrinology, Nanjing First Hospital, Nanjing Medical University, Nanjing, China

**Keywords:** diabetic retinopathy, voxel-mirrored homotopic connectivity, functional magnetic resonance imaging, functional network, functional connectivity

## Abstract

**Background:** Accumulating lines of evidence demonstrated that diabetic retinopathy (DR) patients trigger abnormalities in brain’s functional connectivity (FC), whereas the alterations of interhemispheric coordination pattern occurring in DR are not well understood. Our study was to investigate alterations of interhemispheric coordination in DR patients.

**Methods:** Thirty-four DR individuals (19 males and 15 females: mean age: 52.97 ± 8.35 years) and 37 healthy controls (HCs) (16 males and 21 females; mean age: 53.78 ± 7.24 years) were enrolled in the study. The voxel-mirrored homotopic connectivity (VMHC) method was conducted to investigate the different interhemispheric FC between two groups. Then, the seed-based FC method was applied to assess the different FCs with region of interest (ROI) in the brain regions of decreased VMHC between two groups.

**Results:** Compared with HC groups, DR groups showed decreased VMHC values in the bilateral middle temporal gyrus (MTG), lingual/calcarine/middle occipital gyrus (LING/CAL/MOG), superior temporal gyrus (STG), angular (ANG), postcentral gyrus (PosCG), inferior parietal lobule (IPL), and precentral gyrus (PreCG). Meanwhile, altered FC includes the regions of auditory network, visual network, default mode network, salience network, and sensorimotor network. Moreover, a significant positive correlation was observed between the visual acuity-oculus dexter (OD) and zVMHC values in the bilateral LING/CAL/MOG (*r* = 0.551, *p* = 0.001), STG (*r* = 0.426, *p* = 0.012), PosCG (*r* = 0.494, *p* = 0.003), and IPL (*r* = 0.459, *p* = 0.006) in DR patients.

**Conclusion:** Our results highlighted that DR patients were associated with substantial impairment of interhemispheric coordination in auditory network, visual network, default mode network, and sensorimotor network. The VMHC might be a promising therapeutic target in the intervention of brain functional dysfunction in DR patients.

## Introduction

Diabetic retinopathy (DR) is a serious diabetic-related retinal disease (Zhang et al., 2010). The prevalence of DR is 34.6% among diabetes patients worldwide (Yau et al., 2012). The main pathophysiological mechanisms and clinical features of DR are retinal microangiopathy and vision loss. Retinal neurodegeneration is an important pathogenesis of DR and is characterized by glial cell activation and neuronal apoptosis. Furthermore, DR patients were accompanied by cognitive decline. Recently, growing lines of evidence demonstrated that DR is associated with an increased risk of stroke (Hagg et al., 2013; Wong et al., 2020). Accumulating studies demonstrated that DR is closely related to the occurrence of neurodegenerative diseases (Lynch and Abramoff, 2017; Sundstrom et al., 2018). DR patients are at a high risk for neurodegenerative disorder. However, the exact neural mechanisms of neurodegenerative disorder in DR patients remain unclear.

The functional magnetic resonance imaging method provides a new opportunity for non-invasive research on functional and structural changes of brain *in vivo*. Qi et al. (2020) demonstrated that DR had significantly altered brain neural activity changes in the middle occipital gyrus, the left cerebellum, the left inferior temporal gyrus, and the left hippocampus. Dai et al. (2017) reported that DR patients had abnormal functional connectivity (FC) within visual and cognition networks. Meanwhile, Yu et al. (2020) found that the DR group showed different FCs between the V1 and visual-related brain regions. Moreover, Huang et al. (2019) reported that the DR group showed reduction in the efficiency of functional brain network, relative to the healthy control (HC) group. Thus, existing studies are mainly focused on local brain activity and FC in DR patients. The functional architecture of interhemispheric changes in DR patients remains unknown.

Functional homotopy is a basic principle of the brain’s intrinsic functional architecture (Biswal et al., 1995). The consistency of interhemispheric FC is closely related to a variety of neurophysiological function. The corpus callosum plays an important role in the interhemispheric FC, and the corpus callosum dysfunction might lead to impaired interhemispheric coordination within large-scale brain networks (Roland et al., 2017; Bartha-Doering et al., 2021). Recently, the voxel-mirrored homotopic connectivity (VMHC) method quantified the resting-state FC between hemispheres to reflect the interhemispheric coordination pattern. Prior neuroimaging studies revealed that diabetes patients showed abnormal interhemispheric FC, which is closely related to cognitive impairment. Cui et al. (2021) reported that diabetes patients had decreased interhemispheric FC between bilateral lingual gyrus and sensorimotor cortex. Zhang et al. (2021) also found that decreased VMHC values within the default mode network and middle temporal gyrus (MTG) might serve as a sensitive biomarker for cognitive decline. The VMHC method has been successfully applied to investigate the interhemispheric FC changes in anisometropic and strabismic amblyopia (Liang et al., 2017), Alzheimer’s disease (Wang et al., 2015), and alcohol dependence (Guo et al., 2019). DR is a serious complication of diabetes. Thus, the DR patients may be associated with different VMHC patterns from diabetes patients without retinopathy. We hypothesized that DR patients may lead to interhemispheric FC changes within cognition-related brain region.

Based on the above assumptions, our study aimed to determine whether DR patients were associated with abnormal interhemispheric FC. Moreover, the seed-based FC method was used to assess the FC between the seed regions (brain regions of different VMHC values between two groups) and whole brain. Our results might shed new light on underlying neural mechanisms of cognitive decline in DR patients.

## Materials and Methods

### Subjects

Thirty-four DR individuals (19 males and 15 females: mean age: 52.97 ± 8.35 years) and 37 HCs (16 males and 21 females; mean age: 53.78 ± 7.24 years) matched for age, sex, and education participated in the study.

The inclusion criteria of DR patients were as follows (Zhang et al., 2010): fasting plasma glucose ≥7.0 mmol/L, random plasma glucose ≥11.1 mmol/L, or 2-h glucose ≥11.1 mmol/L (Yau et al., 2012); the DR patients showed microaneurysms, hard exudates, and retinal hemorrhages.

All HCs met the following criteria (Zhang et al., 2010): fasting plasma glucose <7.0 mmol/L, random plasma glucose <11.1 mmol/L, and HbA1c <6.5% (Yau et al., 2012); no ocular diseases (Wong et al., 2020); binocular visual acuity ≥1.0; and (Hagg et al., 2013) no ocular surgical history.

### MRI Parameters

MRI scanning was performed on a 3-T MR scanner (Discovery MR 750W system; GE Healthcare, Milwaukee, WI, United States) with an eight-channel head coil. The T1 images have the following parameters: repetition time = 8.5 ms, echo time = 3.3 ms, thickness = 1.0 mm, gap = 0 mm, acquisition matrix = 256 × 256, field of view = 240 mm × 240 mm, and flip angle = 12°; functional images have the following parameters: repetition time = 2,000 ms, echo time = 25 ms, thickness = 3.0 mm, gap = 1.2 mm, acquisition matrix = 64 × 64, field of view = 240 mm × 240 mm, flip angle = 90°, voxel size = 3.6 mm × 3.6 mm × 3.6 mm, and 35 axial slices.

### fMRI Scanning Steps

All subjects were asked to keep in the supine position and lay still, close their eyes without moving and falling asleep, and not to think of anything in particular during MRI scanning (Zhang et al., 2010; Yau et al., 2012). Before MRI scanning, we would tell the whole experimental process in detail to all subjects (Wong et al., 2020). To avoid noise, the subjects wore earplugs during MRI scanning.

### fMRI Data Preprocessing

The preprocessing of fMRI data was performed with the toolbox for Data Processing & Analysis of Brain Imaging (DPABI^1^) (Yan et al., 2016) with the following steps (Zhang et al., 2010): The whole BOLD pictures of each subjects were removed first 10 points picture and then the remained BOLD picture were slice timing, motion corrected and realigned (Yau et al., 2012); normalized data [in Montreal Neurological Institute (MNI) 152 space] were re-sliced (Wong et al., 2020); regress out several covariates [Friston 24-Parameter Model (Friston et al., 1996) includes six head motion parameters, mean framewise displacement (FD), and global brain signal] (Hagg et al., 2013); data with linear trend were removed, and temporal band pass was filtered (0.01–0.1 Hz).

### Voxel-Mirrored Homotopic Connectivity Analysis

According to a previous study (Zuo et al., 2010), VMHC was performed using the DPABI toolkit. The VMHC values were computed as the Pearson correlation between every pair of mirrored interhemispheric voxels’ time series. All VMHC maps were *z*-transformed with Fisher’s *r*-to-*z* transformation.

### Resting State Functional Connectivity Analysis

After fMRI data were normalized, the fMRI map was smoothed with a 6-mm full-width-half-maximum Gaussian kernel, several covariates were regressed out, and temporal band pass was filtered (0.01–0.1 Hz). The brain regions of different VMHC values were defined as regions of interest (ROIs). Then, the resting state functional connectivity (RSFC) method was applied to assess the FC between the seed regions and whole brain to obtain FC maps.

### Statistical Analysis

The χ^2^ test and independent-samples *t*-test were applied to compare behavioral data between two groups.

One-sample *t*-test was conducted to assess intra-group patterns of zVMHC maps between two groups. Two-sample *t*-tests were conducted to assess different zVMHC and zFC between two groups (two-tailed, voxel-wise *p* < 0.01, GRF theory connected, cluster level, *p* < 0.05).

Pearson correlation was performed to investigate the relationship between the zVMHC values and clinical variables in DR groups.

## Results

### Behavioral Data Comparison

We found the significant difference in best corrected visual acuity (BCVA)-oculus dexter (OD) (*p* < 0.001) and BCVA-oculus sinister (OS) (*p* < 0.001) between two groups. More details are shown in Table 1.

**TABLE 1:** Behavioral data between two groups.

	DR group	HC group	*t*-values	*p*-values
Gender (male/female)	19/15	16/21	N/A	N/A
Age (years)	52.97 ± 8.35	53.78 ± 7.24	−0.412	0.681
Duration of diabetes (years)	10.44 ± 6.03	N/A	N/A	N/A
BCVA-OD	0.47 ± 0.27	1.23 ± 0.23	−12.593	<0.001
BCVA-OS	0.44 ± 0.31	1.22 ± 0.23	−12.121	<0.001
HbA1c (%)	7.69 ± 2.44	N/A	N/A	N/A

*Behavioral data (means ± SD). DR, diabetic retinopathy; HC, healthy control; N/A, not applicable; BCVA, best corrected visual acuity; OD, oculus dexter; OS, oculus sinister; Hb, glycosylated hemoglobin.*

### Voxel-Mirrored Homotopic Connectivity Differences

**FIGURE 1:**
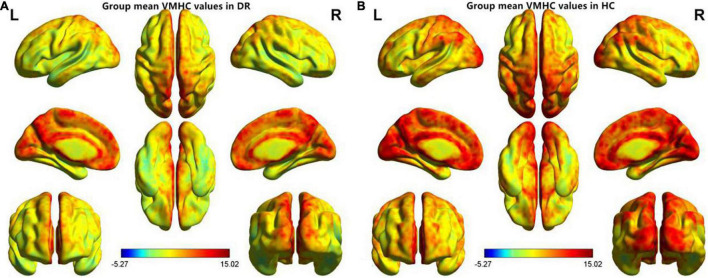
Spatial patterns of VMHC at the group mean level of the DR **(A)** and HC **(B)** groups. VMHC, voxel-mirrored homotopic connectivity; DR, diabetic retinopathy; HC, health controls.

**FIGURE 2:**
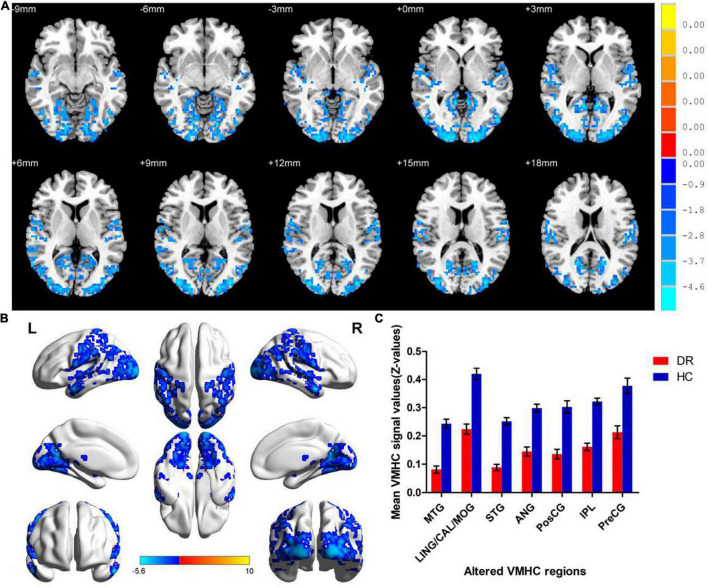
Significant zVMHC maps differences between two groups **(A,B)**. The mean values of altered VMHC values between two groups **(C)**. VMHC, voxel-mirrored homotopic connectivity; DR, diabetic retinopathy; HCs, healthy controls; L, left hemisphere; R, right hemisphere; BA, Brodmann’s area. MTG, middle temporal gyrus; LING/CAL/MOG, lingual/calcarine/middle occipital gyrus; STG, superior temporal gyrus; ANG, angular; PosCG, postcentral gyrus; IPL, inferior parietal lobule; PreCG, precentral gyrus.

Similar spatial patterns of VMHC were found between two groups (Figure 1). Compared with HC groups, DR groups showed decreased VMHC values in the bilateral MTG, lingual/calcarine/middle occipital gyrus (LING/CAL/MOG), superior temporal gyrus (STG), angular (ANG), postcentral gyrus (PosCG), inferior parietal lobule (IPL), and precentral gyrus (PreCG) (Figure 2 and Table 2).

**TABLE 2:** Significant difference in VMHC between two groups.

Condition/brain regions	BA	Peak *t*-scores	MNI coordinates (*x*, *y*, *z*)	Cluster size (voxels)
DR < HC				
MTG	21	−5.141	±54, 0, −21	50
LING/CAL/MOG	18,19	−5.596	±9, −54, 3	1,144
STG	21	−4.643	±60, −9, −9	300
ANG	39	−4.635	±51, −54, 27	95
PosCG	3	−4.140	±45, −15, 39	130
IPL	40	−4.780	±51, −48, 54	312
PreCG	4	−3.683	±21, −30, 72	43

*t: statistical value of peak voxels indicating different VMHC values. VMHC, voxel-mirrored homotopic connectivity; DR, diabetic retinopathy; HCs, healthy controls; MNI, Montreal Neurological Institute; BA, Brodmann’s area; MTG, middle temporal gyrus; LING/CAL/MOG, lingual/calcarine/middle occipital gyrus; STG, superior temporal gyrus; ANG, angular; PosCG, postcentral gyrus; IPL, inferior parietal lobule; PreCG, precentral gyrus.*

### Seed-Based Functional Connectivity Differences

**FIGURE 3:**
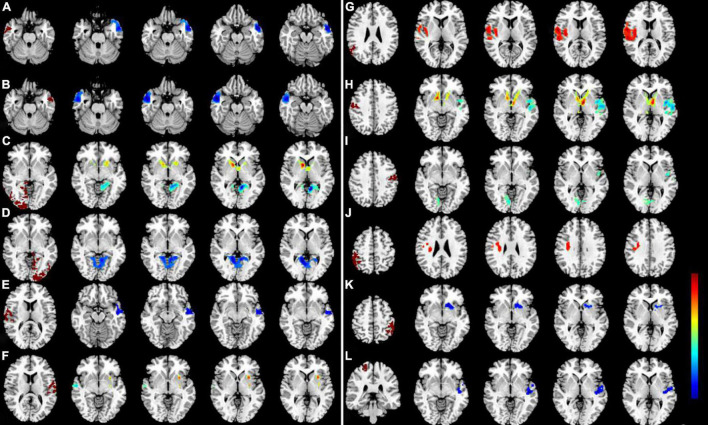
Significant zFC map differences seeded as ROI in different VMHC regions between two groups. Significant different FC values ROI in R-MTG **(A)**, L-MTG **(B)**, R-LING/CAL/MOG **(C)**, R-LING/CAL/MOG **(D)**, R-STG **(E)**, L-STG **(F)**, R-ANG **(G)**, R-PosCG **(H)**, L-PosCG **(I)**, R-IPL **(J)**, L-IPL **(K)**, and R-PreCG **(L)**. VMHC, voxel-mirrored homotopic connectivity; ROI, region of interest; FC, functional connectivity; DR, diabetic retinopathy; HCs, healthy controls; MTG, middle temporal gyrus; LING/CAL/MOG, lingual/calcarine/middle occipital gyrus; STG, superior temporal gyrus; ANG, angular; PosCG, postcentral gyrus; IPL, inferior parietal lobule; PreCG, precentral gyrus.

We investigated resting-state FC seeded as 14 ROIs (seven per hemisphere) with lower VMHC values between two groups (Table 3). This region of altered FC includes the regions of auditory network (MTG and STG), visual network (LING/CAL/MOG), default mode network (ANG and IPL), salience network (insula), and sensorimotor network (PosCG and PreCG) (Figure 3).

**TABLE 3:** Significant difference in FC between two groups in the seed-based analysis.

Seeds	Brain regions	BA	Peak *t*-scores	MNI coordinates (*x*, *y*, *z*)	Cluster size (voxels)
R-MTG	L-MTG	21	–5.6356	−54, 0, −21	335
L-MTG	R-MTG	21	–5.9625	54, 0, −21	355
R-LING/CAL/ MOG	R-CER	–	4.1144	39, −63, −21	275
	L-PUT	–	3.744	−21, 15, −6	96
	L-CAL	18	–5.6603	−9, −54, 3	466
	R-CAU	–	5.2546	12, 6, 6	224
L-LING/CAL/ MOG	B-LING	18	–5.1924	9, −54, 3	599
R-STG	L-MTG	21	–3.8746	−63, −6, −12	163
L-STG	R-TPOsup	38	–4.6008	48, 9, −21	286
	L-PreCG	3	4.2574	−42, −6, 27	96
	L-INS	–	3.7065	−30, 12, 0	65
R-ANG	R-ROL	–	4.1664	60, −3, 12	616
	R-IPL	7	4.5064	18, −45, 51	270
R-PosCG	L-THA	–	4.5859	−6, −6, 0	507
	L-PosCG	4	–5.3769	−57, −15, 18	1,149
	R-PCUN	7	4.4313	15, −63, 42	1,445
L-PosCG	R-LING	18	–3.3128	9, −75, −3	154
	L-ROL	–	–3.6437	−48, 0, 12	70
	L-PosCG	4	–3.8548	−57, −9, 18	187
	R-CUN	19	–3.9295	9, −87, 30	138
	R-PreCG	3	–4.9488	48, −12, 45	481
	L-PCC	–	3.8502	−3, −45, 24	348
R-IPL	R-PosCG	4	4.3822	30, −21, 21	313
L-IPL	L-IFGtriang	–	–4.3103	−33, 24, 27	517
R-PreCG	L-MTG	41	–3.7637	−51, −18, −3	297

*t: statistical value of peak voxels indicating different FC values. VMHC, voxel-mirrored homotopic connectivity; DR, diabetic retinopathy; HCs, healthy controls; MNI, Montreal Neurological Institute; BA, Brodmann’s area; MTG, middle temporal gyrus; LING/CAL/MOG, lingual/calcarine/middle occipital gyrus; STG, superior temporal gyrus; ANG, angular; PosCG, postcentral gyrus; IPL, inferior parietal lobule; PreCG, precentral gyrus; CER, cerebellum_Crus1; PUT, putamen; CAU, caudate; TPOsup, temporal pole (superior); INS, insula; ROL, rolandic operculum; THA, thalamus; PCUN, precuneus; CUN, cuneus; IFGtriang, inferior frontal gyrus (triangular); L, left; R, right; B, bilateral.*

### Receiver Operating Characteristic Curve

**FIGURE 4:**
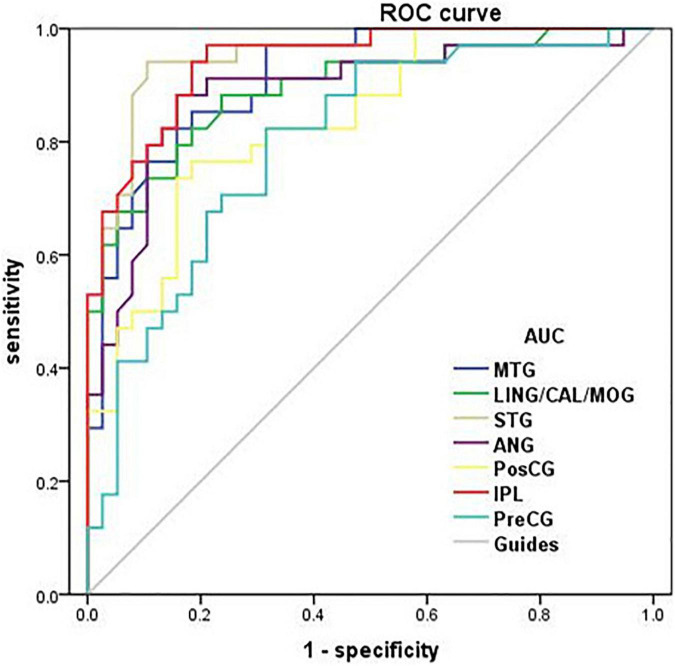
Receiver operating characteristic curve analysis of the mean zVMHC. ROC curve in zVMHC: DR < HC; for MTG, 0.913 (*p* < 0.001; 95% CI: 0.850–0.976); for LING/CAL/MOG, 0.895; for STG, 0.952; for ANG, 0.888; for PosCG, 0.837; for IPL, 0.942; and for PreCG, 0.799. ROC, receiver operating characteristic; AUC, area under the curve; VMHC, voxel-mirrored homotopic connectivity; DR, diabetic retinopathy; HCs, healthy controls; MTG, middle temporal gyrus; LING/CAL/MOG, lingual/calcarine/middle occipital gyrus; STG, superior temporal gyrus; ANG, angular; PosCG, postcentral gyrus; IPL, inferior parietal lobule; PreCG, precentral gyrus.

The area under curve (AUC) for zVMHC was as follows: DR < HC, for MTG, 0.913; for LING/CAL/MOG, 0.895; for STG, 0.952; for ANG, 0.888; for PosCG, 0.837; for IPL, 0.942; and for PreCG, 0.799 (Table 4 and Figure 4).

**TABLE 4:** Receiver operating characteristic curve analysis.

Condition/brain regions		AUC	*p*-values	95% CI
DR < HC	MTG	0.913	*p* < 0.001	0.850–0.976
DR < HC	LING/CAL/MOG	0.895	*p* < 0.001	0.821–0.969
DR < HC	STG	0.952	*p* < 0.001	0.906–0.998
DR < HC	ANG	0.888	*p* < 0.001	0.806–0.969
DR < HC	PosCG	0.837	*p* < 0.001	0.746–0.927
DR < HC	IPL	0.942	*p* < 0.001	0.893–0.991
DR < HC	PreCG	0.799	*p* < 0.001	0.697–0.902

*ROC, receiver operating characteristic; CI, confidence interval; AUC, area under the curve; MTG, middle temporal gyrus; LING/CAL/MOG, lingual/calcarine/middle occipital gyrus; STG, superior temporal gyrus; ANG, angular; PosCG, postcentral gyrus; IPL, inferior parietal lobule; PreCG, precentral gyrus.*

### Pearson Correlation Analysis

**FIGURE 5:**
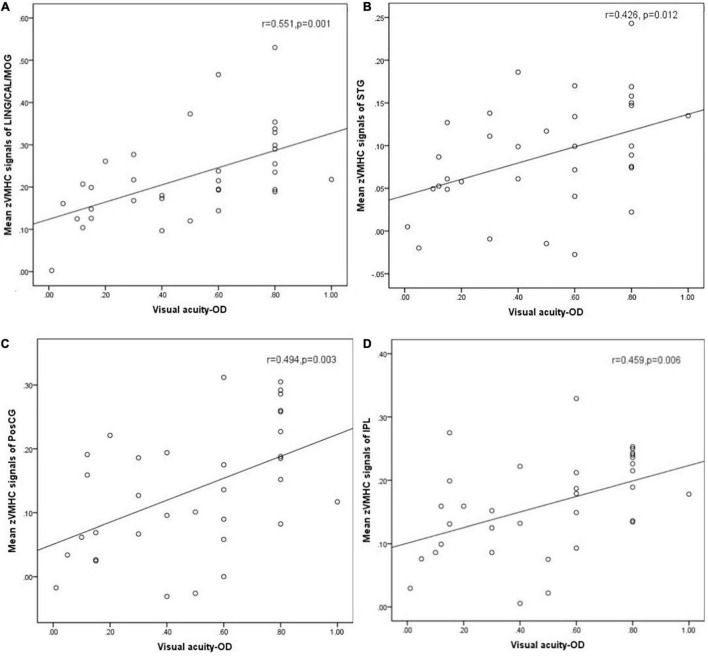
Correlation was found between the visual acuity-OD and zVMHC values in the bilateral LING/CAL/MOG (*r* = 0.551, *p* = 0.001), **(A)** STG (*r* = 0.426, *p* = 0.012), **(B)** PosCG (*r* = 0.494, *p* = 0.003), **(C)** and IPL (*r* = 0.459, *p* = 0.006) **(D)** in DR patients. DR, diabetic retinopathy; VMHC, voxel-mirrored homotopic connectivity; OD, oculus dexter; LING/CAL/MOG, lingual/calcarine/middle occipital gyrus; STG, superior temporal gyrus; PosCG, postcentral gyrus. IPL, inferior parietal lobule.

A significant positive correlation was found between the visual acuity-OD and zVMHC values in the bilateral LING/CAL/MOG (*r* = 0.551, *p* = 0.001), STG (*r* = 0.426, *p* = 0.012), PosCG (*r* = 0.494, *p* = 0.003), and IPL (*r* = 0.459, *p* = 0.006) in DR patients (Figure 5).

## Discussion

The VMHC method is a sensitive and high-resolution resting-state fMRI technology, which is applied to assess the FC between two cerebral hemispheres. In our study, we found that DR patients had decreased interhemispheric FC within auditory network, visual network, default mode network, and sensorimotor network. A significant positive correlation was observed between the visual acuity-OD and zVMHC values in the bilateral LING/CAL/MOG (*r* = 0.551, *p* = 0.001), STG (*r* = 0.426, *p* = 0.012), PosCG (*r* = 0.494, *p* = 0.003), and IPL (*r* = 0.459, *p* = 0.006) in DR patients.

We found that DR groups showed decreased VMHC values in the LING/CAL/MOG, which was located in the visual network. The main pathological changes of DR include microaneurysms, hemorrhages, hard exudates and macular edema, and even vitreous hemorrhage and tractional retinal detachment in the late stage. These pathological changes of the retina can lead to vision loss. Meanwhile, DR not only causes retinal changes, but also leads to structural and functional abnormalities in visual pathways. Li Y. M. et al. (2018) demonstrated that DR patients were accompanied by abnormal white matter integrity in visual pathway. Qi et al. (2021) found that DR patients had a significant lower FC within the visual cortex, relative to HC group. Corduneanu et al. (2019) also reported that DR patients had latency of waves on the visual evoked potential pathway wave relative to the HC group. Moreover, Wang et al. (2017) found that the increased apparent diffusion coefficient values of the visual cortex were observed in patients with proliferative and non-proliferative DR. With the support of these findings, we also demonstrated that DR patients showed decreased interhemispheric FC within the visual network, which might reflect the impaired interhemispheric coordination in processing of visual information in DR patients. We speculated that visual loss might contribute to the reduced interhemispheric FC within the visual network.

Another important finding is that DR patients had reduced interhemispheric FC within the auditory network. Prior studies demonstrated that diabetes patients were accompanied by hearing impairment. Li J. et al. (2018) reported that diabetes patients had bilateral sensorineural hearing loss, especially at high frequencies. AlJasser et al. (2020) also found that the T1DM group showed significantly reduced frequency-following response to both temporal envelope and temporal fine structure. Braite et al. (2019) reported that T1DM patients were associated with auditory efferent pathway dysfunction. Meanwhile, Willette et al. (2013) found that diabetes patients showed medial temporal lobe atrophy and decreased white matter in the left temporal lobe (Northam et al., 2009). Chen et al. (2012) demonstrated that T2DM patients were accompanied by gray matter atrophy in the temporal gyri. With these findings, our study revealed that DR patients had reduced interhemispheric FC within auditory network, which might reflect the impaired auditory function in DR patients. However, the exact neural mechanism of auditory function dysfunction in DR patients remain unclear.

In addition, we found that DR patients had reduced interhemispheric FC within the sensorimotor network. The sensorimotor network plays an important role in motor control and sensory function. Previous studies demonstrated that diabetic peripheral neuropathy patients were associated with sensorimotor dysfunction (Khan et al., 2020; Van Eetvelde et al., 2020). Zhang et al. (2020) demonstrated that diabetic peripheral neuropathy patients had abnormal gray matter in pre- and PosCG relative to health controls. Hansen et al. (2019) found that diabetes patients’ diabetes had 9.3% lower ratio of *N*-acetyl aspartate/creatine (NAA/cre) in the parietal region including the sensorimotor fiber tracts. Meanwhile, van Duinkerken et al. (2017) also reported that one diabetes mellitus patient had decreased FC within the sensorimotor network. Thus, we also found that DR patients had reduced interhemispheric FC within the sensorimotor network, which might indicate the impaired sensorimotor function.

We found that DR patients had widespread decreased interhemispheric FC within the default mode network (DMN). Previous neuroimaging studies demonstrated that diabetes patients were associated with DMN dysfunction, which is closely related with cognitive decline (Cui et al., 2015; Chen et al., 2016; Yang et al., 2016; Tan et al., 2019). In our study, we found that DR patients had widespread decreased interhemispheric FC within the DMN, which reflect the interhemispheric FC dysfunction in DMN functional communication.

Some limitations should be acknowledged in this study. First, VMHC results based on blood oxygenation level-dependent (BOLD) signals would still be affected by physiological noise. Second, BOLD signals may be influenced by the subject’s subjective thinking. We asked subjects not to think of anything in particular during MRI scanning in the study. Third, the exact neural mechanisms of interhemispheric FC dysfunction are still unclear, and multimodal neuroimaging and machine learning algorithms should be combined to evaluate the neural mechanisms changes in DR patients in future studies.

In conclusion, our results highlighted that DR patients are associated with substantial impairment of interhemispheric coordination in auditory network, visual network, DMN, and sensorimotor network. These findings shed a new light on the neural mechanism of DR patients.

## Data Availability Statement

The raw data supporting the conclusions of this article will be made available by the authors, without undue reservation.

## Ethics Statement

The studies involving human participants were reviewed and approved by the Jiangxi Provincial People’s Hospital Affiliated to Nanchang University. The patients/participants provided their written informed consent to participate in this study.

## Author Contributions

SW, WX, and YZ contributed to the data collection, statistical analyses, wrote the manuscript, design the protocol, and MRI analysis. SW and WX designed the study and oversaw all clinical aspects of study conduct and manuscript preparation. All authors contributed to the article and approved the submitted version.

## Conflict of Interest

The authors declare that the research was conducted in the absence of any commercial or financial relationships that could be construed as a potential conflict of interest.

## Publisher’s Note

All claims expressed in this article are solely those of the authors and do not necessarily represent those of their affiliated organizations, or those of the publisher, the editors and the reviewers. Any product that may be evaluated in this article, or claim that may be made by its manufacturer, is not guaranteed or endorsed by the publisher.

## Footnotes

1
http://www.rfmri.org/dpabi
