# Corrigendum: Aberrant Interhemispheric Functional Connectivity in Diabetic Retinopathy Patients

**DOI:** 10.3389/fnins.2022.860597

**Published:** 2022-02-15

**Authors:** Song Wan, Wen Qing Xia, Yu Lin Zhong

**Affiliations:** ^1^Department of Ophthalmology, Jiangxi Provincial People's Hospital, Nanchang, China; ^2^Department of Endocrinology, Nanjing First Hospital, Nanjing Medical University, Nanjing, China

**Keywords:** diabetic retinopathy, voxel-mirrored homotopic connectivity, functional magnetic resonance imaging, functional network, functional connectivity

In the published article, there was an error in affiliation 1. Instead of “Department of Ophthalmology, Jiangxi Provincial People's Hospital Affiliated to Nanchang University, Nanchang, China,” it should be “Department of Ophthalmology, Jiangxi Provincial People's Hospital, Nanchang, China.”

In the published article, there was also an error in the affiliation for ^**^Wen Qing Xia^**^. Instead of ^**^Wen Qing Xia^1, 2^†^**^ it should be ^**^Wen Qing Xia^2^†^**^.

The authors apologize for this error and state that this does not change the scientific conclusions of the article in any way. The original article has been updated.

## Publisher's Note

All claims expressed in this article are solely those of the authors and do not necessarily represent those of their affiliated organizations, or those of the publisher, the editors and the reviewers. Any product that may be evaluated in this article, or claim that may be made by its manufacturer, is not guaranteed or endorsed by the publisher.

